# Microglial Replacement Reverses Age-Associated Epigenetic Modifications Despite Accelerating Epigenetic Age

**DOI:** 10.14336/AD.2025.1066

**Published:** 2026-10-11

**Authors:** Maria Arbaizar-Rovirosa, Raúl F. Pérez, Alfonso Peñarroya, Mattia Gallizioli, Mario F. Fraga, Anna M. Planas

**Affiliations:** ^1^Cerebrovascular Research Laboratory, Instituto de Investigaciones Biomédicas de Barcelona (IIBB), Consejo Superior de Investigaciones Científicas (CSIC), Barcelona, Spain.; ^2^Institut d’Investigacions Biomèdiques August Pi i Sunyer (IDIBAPS), Barcelona, Spain.; ^3^Departamento de Bioquímica y Biología Molecular, Facultad de Veterinaria, Universidad Complutense de Madrid, Madrid, Spain.; ^4^Cancer Epigenetics and Nanomedicine Laboratory, Centro de Investigación en Nanomateriales y Nanotecnología-Consejo Superior de Investigaciones Científicas (CINN-CSIC), Universidad de Oviedo, Oviedo, Spain.; ^5^Instituto de Investigación Sanitaria del Principado de Asturias (ISPA), Oviedo, Spain.; ^6^Servicio de Reumatología, Hospital Universitario, Madrid, Spain.; ^7^Grupo de Investigación en Enfermedades Inflamatorias y Autoinmunes, Instituto de Investigación Hospital, Madrid, Spain.; ^8^Instituto Universitario de Oncología del Principado de Asturias (IUOPA), Universidad de Oviedo, Spain.; ^9^Centro de Investigación Biomédica en Red de Enfermedades Raras (CIBERER), Instituto de Salud Carlos III (ISCIII), Madrid, Spain.; ^10^Departamento de Biología de Organismos y Sistemas, Área de Fisiología Vegetal, Universidad de Oviedo, Oviedo, Spain.

**Keywords:** DNA methylation, microglia, repopulation, aging, stroke, epigenetic clock

## Abstract

Microglial replacement is emerging as a promising concept for treating age-related disorders, but effects on epigenetic age remain unclear. Here, we examined DNA methylation dynamics in microglia from young and old mice and evaluated how ischemic stroke and microglial depletion/repopulation (D/R) influence their epigenetic landscape. Using epigenetic clocks, we confirmed that old microglia display an aged DNA methylation profile, consistent with functional decline. Both stroke and microglial D/R induced an acceleration of epigenetic age, likely reflecting proliferative stress associated with these conditions. However, genome-wide methylation profiling using DNA methylation arrays revealed that microglial repopulation also reversed a large fraction of age-associated DNA methylation changes, particularly within pathways related to immune activation and inflammatory responses. These findings suggest that microglial D/R, though linked to epigenetic age acceleration, leads to the widespread reversal of aging-associated DNA methylation changes, which may help explain the beneficial outcomes observed after microglial replacement. Overall, our results highlight the complexity of interpreting epigenetic age measures and underscore the potential of microglial replacement strategies for brain rejuvenation.

## INTRODUCTION

Microglia are critically involved in aging and age-associated diseases. Aging imposes changes in the transcriptional profile of microglia governed by epigenetic alterations [1] resulting in cellular senescence [2]. In the elderly, microglia exhibit a primed pro-inflammatory phenotype [3] and display age-related transcriptomic signatures indicative of dysfunctional states [4, 5]. Similarly, following acute brain injury, microglia from aged mice display exaggerated inflammatory responses [6, 7]. Strategies of microglia replacement are under study to enhance microglial function and restore homeostasis under disease conditions [8, 9]. Remarkably, microglia replacement has been applied to humans with microgliopathy, yielding promising results [10]. Targeted microglia depletion can be achieved through pharmacological inhibition of colony-stimulating factor-1 receptor (CSF1R) [11]. Transient microglia depletion in adult mice is not pathogenic, and, after treatment cessation, microglia spontaneously repopulate the brain, driven by proliferation of residual microglia [12].

Microglial depletion and repopulation (D/R) in aged mice restored cognitive, synaptic, and neuronal function, and induced signs of neuronal rejuvenation, including transcriptional, morphological, and functional changes [13]. While D/R only partially restored aged microglial transcriptional profiles, it fully reversed their lipid-laden phenotype [14]. Moreover, microglia D/R in aged mice attenuated the exaggerated inflammatory transcriptional profile and lipid accumulation characteristic of aged microglia following stroke, and improved motor function, suggesting a shift toward a more youthful microglia phenotype [4]. Likewise, microglia replacement attenuated neuroinflammation, and reduced neurological deficits and brain edema after intracerebral hemorrhage in aged mice [15]. However, the specific changes in repopulated microglia underlying their improved function versus the original aged microglia remain unknown. Age drives progressive alterations in DNA methylation (DNAm) that can advance the epigenetic age, often correlating with functional decline. Microglia are long-lived cells that self-renew throughout the lifespan [16], accumulating age-related changes in their epigenetic landscape over time [1]. We hypothesized that microglial D/R can modify the epigenetic signature of newly formed microglia, giving them a younger epigenetic profile. The biological age of cells, tissues, and organisms can be estimated based on specific DNA methylation patterns described through epigenetic clocks [17]. Moreover, pathological states can accelerate epigenetic age beyond chronological age, a phenomenon referred to as epigenetic age acceleration, whereas interventions that restore molecular features of youth are generally described as rejuvenation. The aim of this work was to identify the epigenetic age of microglia in young and old mice, and to study the effects of stroke and microglial D/R in old mice on this parameter.

## MATERIALS AND METHODS

### Mice

C57BL/6 adult young (3-4 months) mice and old (21-22 months) mice were obtained from Janvier (Lyon, France) or Envigo (Amsterdam, The Netherlands). Mice were maintained at the animal facility at the School of Medicine or Faculty of Psychology of the University of Barcelona (UB) under standardized environmental conditions, a 12 h dark/light cycle, and ad libitum access to food and water. Both male and female mice were used, but sexes were not mixed in all the experiments. Animal work was conducted following the Catalan and Spanish laws (Real Decreto 53/2013) and the European Directives. All experiments were conducted with the approval of the ethical committee (Comité Ètic d’Experimentació Animal, CEEA) of Universitat de Barcelona, and the local regulatory bodies of the Generalitat de Catalunya, and in compliance with the NIH Guide for the Care and Use of Laboratory Animals.

### Brain ischemia model

Cerebral ischemia was induced in mice by transient occlusion of the right middle cerebral artery (MCAo) with the intraluminal technique, as previously described [18]. In brief, anesthesia was induced with 5% isoflurane in a mixture of 30% O_2_ and 70% N_2_O and was maintained with 1.5% isoflurane in the same mixture using a facial mask. Cerebral blood flow was monitored with laser Doppler flowmetry during the procedure to guide the filament insertion and monitor perfusion. A laser Doppler (Perimed, AB, Järfälla, Sweden) was placed on the territory of the right MCA (2.5mm lateral from Bregma) with the help of a Doppler holder attached to the skull using cyanoacrylate. With the animal in supine position on a heated surgery table (37°C), a longitudinal cut was made in the ventral midline of the neck and the submaxillary glands and the omohyoid and sternothyroid muscles were retracted, exposing the common carotid artery (CA). A monofilament (#701912PK5Re, Doccol Corporation, Sharon, MA) was introduced through the right external CA, towards the internal CA, up to the level where the MCA branches out. We allowed the mice to wake up and kept them on the heated blanket. After 45 min of arterial occlusion, mice were anesthetized again, the filament was cautiously removed, and the suture of the common CA was removed to allow reperfusion. Mice received analgesia (buprenorphine, 150 µl, 0.015 mg/mL via s.c.) and were kept on a thermal blanket at 37°C for 1 h after surgery.

Pre-established exclusion criteria were either unsuccessful MCAo resulting in no infarction (assessed by MRI) or technical surgical complications. Two weeks post-ischemia mice were euthanized, and brains were collected.

### Magnetic resonance imaging

The brain lesions were imaged using magnetic resonance imaging (MRI) in a 7.0 T BioSpec 70/30 horizontal animal scanner equipped with a 12-cm inner diameter actively shielded gradient system (400 mT/m). The receiver coil was a phased array surface coil for mouse brain (Bruker BioSpin, Ettlingen, Germany). Both 4 days and 14 days after induction of ischemia, the brain was studied with T2w turbo RARE fast spinecho MRI sequences with one effective echo time (ET) = 33 ms, slice thickness = 0.5 mm, repetition time (TR) = 2,336 ms, field of view = 20 × 20 mm2, matrix size = 256 pixels, and in-plane spatial resolution = 0.078 mm. Images were obtained with ParaVision 6.0 software (Bruker BioSpin, Ettlingen, Germany). Image analysis was carried out using Fiji 2.2.0-2.3.0.

### Drug Treatment

For microglia depletion, mice received the CSF1R inhibitor PLX5622 (MedChemExpress #HY-114153) following a previously reported protocol [7]. PLX5622 was mixed into AIN-76A rodent diet at 1,200 ppm (Research Diets, NJ, USA; supplied by Brogaarden, Denmark). Treatment controls received AIN-76A drug-free standard chow for the same period. Both diets were given in parallel in groups of five animals per cage. Mice received diet *ad libitum*. After two weeks of treatment, the PLX5622 diet was discontinued and replaced with standard chow for seven days. At this point, stroke was induced in the designated groups of mice by brain ischemia. Brains were collected three weeks after discontinuing the PLX5622 diet to study the effects of microglial repopulation.

### Brain tissue processing and microglial isolation by fluorescence-activated cell sorting

Mice were anesthetized with isoflurane and euthanized by cervical dislocation. Brains were collected in 50 mL falcon tubes with cold HBSS buffer (w/o Ca^2+^ and Mg^2+^; #14175-053, Thermo Fisher Scientific) and placed on ice. The forebrain was dissected with a scalpel, discarding the cerebellum and the olfactory bulbs. The ipsilateral and contralateral hemispheres were separated, minced into small pieces and placed into gentleMACS™ C-Tubes (#130-096-334, Miltenyi Biotec). The brain tissue was enzymatically dissociated using the Neural Tissue Dissociation Kit (P) (#130-092-628, Miltenyi Biotec). The gentleMACSTM Octo Dissociator (#130-096-427, Miltenyi Biotec) was used for mechanical dissociation steps following the Neural Tissue Dissociation Kit (P) manufacturer protocol for dissociation with heaters (1× m_Brain_1 program and 1× ABDK_37C program by Miltenyi).

The digested tissue was then filtered through a 70 µm cell strainer (#352350, Falcon), previously humidified, with Hanks’ balanced salt solution (HBSS) with Ca^2+^ and Mg^2+^ (#14025-092, Thermo Fisher Scientific). Then, cells were separated from myelin by an immunomagnetic separation method. Brain cells were incubated with Myelin Removal Beads II (#130-096-733, Miltenyi Biotec) and then passed through LS Columns (#130-042- 401, Miltenyi Biotec) held onto the OctoMACS Separator (#130-091- 051, Miltenyi Biotec) and to the MACS MultiStand (#130-042-303, Miltenyi Biotec). The negative fraction was collected to continue with the staining protocol.

Unspecific binding of antibodies was blocked by previous incubation for 10 min with anti-CD16/CD32 (Fc block, clone 2.4G2; #55314, BD Pharmingen) in Fluorescence-Activated Cell Sorting (FACS) buffer at 4°C. Live/dead Aqua cell stain (#L34957, Thermo Fisher Scientific) was used to determine cell viability. Cells were then incubated with the following primary antibodies during 30 min at 4°C: CD11b (clone M1/70, AF647, #557686, BD Pharmigen), CD45 (clone 30-F11, FITC, #553080, BD Pharmingen) and CD31 (clone 390, PE-Cy7, #25-0311-82, eBioSciences). After washing with FACS Stain Buffer (#554656, BD Biosciences), cells were sorted in a FACS Aria II or FACS Aria SORP sorter (BD Biosciences) using FacsDiva software (version 5, BD Biosciences, San Jose, CA, USA).

Microglial cells were collected in sterile 1.5 mL Eppendorf tubes previously coated with FBS (#A3160501, Thermo Fisher Scientific) and 200 µL of cold dPBS (#14190-094, Thermo Fisher Scientific). As fast as possible, cells were centrifuged, the supernatant was removed, and cells were snap-frozen in dry ice. Data analyses were performed with FlowJo software (version 10, FlowJo LLC, Ashland, OR, USA).

### Bisulfite pyrosequencing for epigenetic age estimation

Bisulfite pyrosequencing was used to estimate the Wagner mouse epigenetic clock as previously described [19]. In brief, DNA was extracted with a standard phenol-chloroform protocol. Bisulfite conversion was performed with the EZ DNA methylation-gold kit (Zymo Research). After PCR amplification, pyrosequencing was performed using PyroMark Q24 reagents and a vacuum prep workstation, equipment and software (Qiagen). Primers used were those described by Han Y and colleagues [19]. Linear mixed-effects models for comparing epigenetic age values across groups were fit with the R package *lme4* (v1.1-35.5) [20] and p-values were computed using Satterthwaite’s method via the R package *lmerTest* (v3.1-3) [21].

### Mouse Methylation BeadChips

Whole-genome DNA methylation (DNAm) levels were assessed through Illumina Infinium Mouse Methylation BeadChips. In short, DNA was extracted with a standard phenol-chloroform protocol. Quantity and integrity were assessed through fluorimetry (Qubit) and agarose gel electrophoresis, respectively. Bisulfite conversion was performed with the EZ DNA methylation kit (Zymo Research). Samples were then processed and hybridized to BeadChips following the Illumina Infinium Mouse Methylation protocol.

### Preprocessing of DNA methylation array data

All data preprocessing and analyses were carried out using the statistical software R (v4.3.1). IDAT files were imported and processed using the R package *sesame* (v1.20.0) [22]. Intensity measurements were transformed into beta values using the openSesame function with recommended settings for mouse arrays (“prep” argument set to “TQCDPB”), which includes non-linear dye bias correction and noob background correction [23]. Beta values for probes with multiple technical replicates were averaged. Sex and mouse strain were inferred to confirm sample identity. Additionally, the following probes were filtered out: those masked by the SeSAMe pipeline (poor-design probes and probes with detection p-values > 0.05 in any sample) [22], those flagged or with missing values in the “MFG_Change_Flagged” column from the Mouse Methylation BeadChip Manifest File v1.0 A2 (https://support.illumina.com/array/array_kits/infinium-mouse-methylation-beadchip-kit.html), non-CpG probes, probes with missing values in any sample, and probes mapping to the mitochondrial chromosome. The final number of profiled CpG sites was 259,148.

### Genomic annotation of CpG sites

CpG sites were annotated to genes and genomic locations using the Gencode mouse M25 release comprehensive gene annotation (mm10) via the R package *ChIPseeker* (v1.38.0) [24]. Promoters were defined as 2kb upstream of transcription start sites. In addition, CpG sites were annotated to CpG islands using the mm10 unmasked island annotation from the UCSC Table browser [25]. CpG island shores and shelves were defined as regions flanking CpG islands by 2 kb and 4 kb, respectively.

### Differential methylation analyses

Differential comparisons were performed using M-values [26]. The R package *limma* (v3.58.1) [27] was used to build linear models and carry out comparisons using an empirical Bayes moderated t-test. Differentially methylated probes (DMPs) were defined as those with FDR-adjusted p-value < 0.05 and a change in DNAm > 5%.

### Epigenetic age estimation using array data

Various epigenetic clocks were computed using the array data. The Wagner mouse blood epigenetic clock [19], which was developed for pyrosequencing and measures 3 CpG sites, was estimated by using the DNAm values for the closest CpG sites measured by the array. The following CpG sites were used: 1) cg31044702, which is the exact *Prima1* site used in the original clock; 2) cg46095458, which is 5 bp upstream of the *Hsf4* site in the original clock; 3) cg39241924, which is 204 bp downstream of the *Kcns1* site in the original clock.

The Reik mouse multi-tissue epigenetic clock [28], which was developed using reduced representation bisulfite sequencing (RRBS) technology and measures 329 CpG sites, was computed by using the array DNAm values for the CpG sites directly intersecting or at 1 bp distance of the original clock sites. A total of 142 CpG sites were available in the array, and the rest was imputed with a beta value of 0 across all samples. Alternative imputation strategies were explored, in which missing beta values were either sampled from the rest of the array or from a bimodal beta distribution (a 1:1 mixture of Beta (1,10) and Beta (10,1)). Across 1,000 permutations, the average clock values showed a Pearson correlation >0.99 with the beta = 0 imputation strategy. However, individual clock estimates were highly variable, with a median correlation of 0.3 across permutations, highlighting the uncertainty introduced by imputation. Despite this variability, group differences (young vs. old, and old vs. old D/R) remained statistically significant.

The proliferative history epiCMIT clock [29] was developed in human blood samples by tracking average DNAm values at a large number of CpG sites (N = 1,348). To adapt the clock to mouse array data, the human hg19 coordinates were lifted to mm10 with the *easylift* R package (v1.0.0) which internally uses the liftOver functionality implemented in the *rtracklayer* R package (v1.62.0) [30, 31] using the appropriate chain file (https://hgdownload.soe.ucsc.edu/downloads.html).

Then, mouse array CpGs within 250 bp of the lifted clock regions were used for the computation of epiCMIT: 127 sites were finally used for epiCMIT hyper, which represent sites gaining DNAm with proliferative history and 147 sites for epiCMIT hypo, which represent sites losing DNAm with proliferative history. Finally, epiCMIT hyper was computed as the average of the hypermethylation sites, and epiCMIT hypo was computed as 1 minus the average of the hypomethylation sites.

### Pathway enrichment analyses

To perform pathway enrichment analyses using sets of CpGs, these were first annotated to genes as described above. Only those genes from non-intergenic CpGs were retained. Then, the R package *goseq* (v1.54.0) [32] was used to perform gene set enrichment using different databases from MSigDB [33] including Gene Ontology [34] and Hallmark [35], which were accessed and mapped to mouse gene IDs with the R package *msigdbr* (v7.5.1). The enrichments were adjusted to account for the differing numbers of CpGs profiled per gene, which is a potential source of bias. Significantly enriched pathways were defined as those with an FDR-adjusted overrepresentation p-value < 0.05. For inspection of the most relevant results, only those pathways with adjusted p-value < 0.05, odds ratio (OR) > 1.5, more than 15 and less than 750 genes were considered.

### Enhancer enrichment analyses

We accessed cell type-specific enhancer location data from the EnhancerAtlas 2.0 web server (www.enhanceratlas.org/) [36]. The *easylift* R package (v1.0.0) was used to transform mm9 coordinates into mm10 coordinates using the appropriate chain file (https://hgdownload.soe.ucsc.edu/downloads.html).

Then, array CpGs were annotated to enhancer locations when a direct overlap was observed.

## RESULTS

We evaluated the effects of ischemic stroke on the epigenetic age status of microglia of young (12wk, N=22) and old (91wk, N=20) female mice and tested the effect of microglial depletion, induced by the CSF1R inhibitor PLX5622, followed by repopulation (D/R, 91wk, N=18) (Fig. 1A, Methods). Evaluation of epigenetic age by bisulfite pyrosequencing using the Wagner epigenetic clock [19] confirmed that this blood-based predictor distinguished microglia from young and old subjects (t-test p=0.035, Fig. 1B). Stroke increased the microglial epigenetic age across all subjects (linear mixed-effects model p=0.0256, adjusted for age and subject, Fig. 1C), though differences between individual age groups did not reach statistical significance (paired t-test, Fig. 1C). These findings are consistent with human data whereby stroke is associated with increased epigenetic age [37, 38]. Unexpectedly, microglial D/R induced strong epigenetic age acceleration across all samples (linear mixed-effects model p<0.001, adjusted for ischemia and subject, Fig. 1D), and within each group (t-test both p<0.001, Fig. 1D). A mixed-effects model using all samples (N=60) including age, stroke, and microglia D/R groups, demonstrated that all three variables were significantly associated with increased epigenetic age of microglia (all p<0.001). These results contrast previous reports of microglial D/R alleviating aging-associated phenotypes [7, 8, 13, 39], though repeated cycles of microglia D/R induce an aged-like phenotype due to proliferative stress [1]. Thus, the epigenetic age acceleration following microglial D/R may be due to the increased proliferation inherent to the process of microglial repopulation.

To deepen our understanding of the epigenetic processes associated with microglial D/R, we conducted a study with male mice including young (N=4), old (N=4) and old-D/R (N=4) mice (Fig. 1E). We performed genome-wide characterization of DNAm using mouse methylation arrays, profiling the levels of 259,148 CpG sites across all subjects (see Methods). Principal component analysis revealed a clear segregation of the sample groups (Fig. 1F), indicating global differences across their DNA methylome. Estimating the Wagner epigenetic age using the closest available CpGs in the array showed a significant difference between microglia of young and old male mice (t-test p=0.003, Fig. 1G). Additionally, we detected a non-significant trend for increased epigenetic age in the old D/R group (t test p=0.140, Fig. 1G). By implementing a sequencing-based multi-tissue epigenetic clock from the Reik lab [28], for which we had measurements at 142 related CpG sites in our array (43%), we observed increased epigenetic age in old microglia (t-test p=0.001, Fig. 1H), and an additional gain in D/R microglia (t-test p=0.010, Fig. 1H).

To test the potential relationship between the observed epigenetic age acceleration and proliferation in our study system, we adapted epiCMIT, a human blood-based epigenetic predictor of proliferative history [29] to our mouse array data. epiCMIT quantifies DNAm in biologically-defined regions with known replication-dependent changes [29], and the chromatin context of aging epigenetic alterations is conserved between human and mouse [40]. We detected an increase in proliferative history with aging and a trend for repopulated microglia for the hypermethylation epiCMIT clock (t-test p=0.024 and 0.151 for aging and D/R, respectively, Fig. 1I). Furthermore, we found a particular increase in proliferative history in the D/R group for the hypomethylation epiCMIT clock (t-test p=0.002, Fig. 1J). These results suggest that the epigenetic age acceleration following microglial D/R may be partially linked to the increased proliferation required to repopulate the niche left empty after microglial depletion.

**Figure 1.**
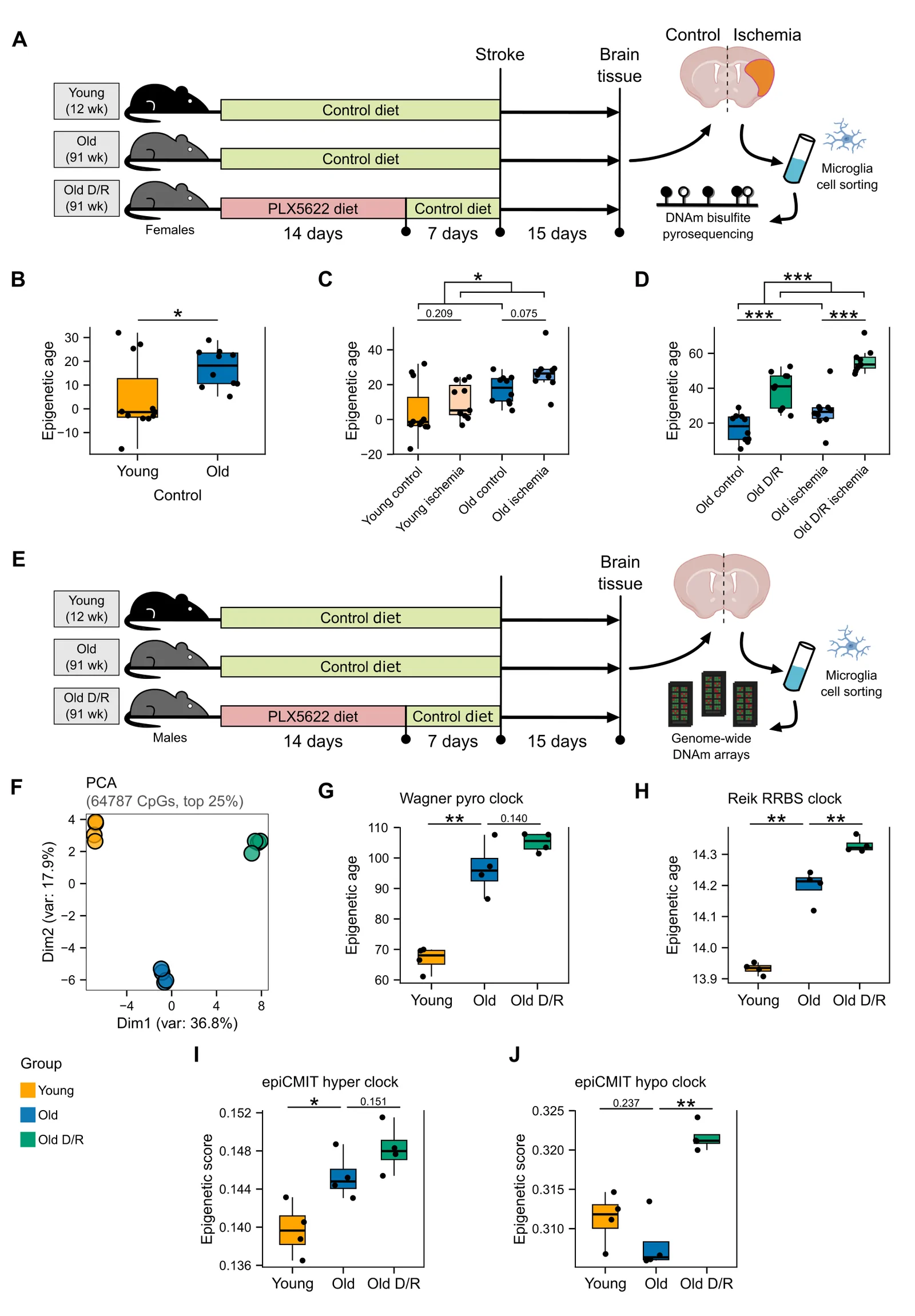
**Increased epigenetic aging in microglia in response to stroke or repopulation**. (**A**) Experimental design involving stroke in young and old female mice, and PLX5622-mediated microglial depletion and repopulation (D/R) in old mice. (**B-D**) Epigenetic age values measured by the pyrosequencing-based Wagner epigenetic clock in the different groups. (**E**) Experimental design involving PLX5622-mediated microglial D/R in old male mice. (**F**) Principal component analysis (PCA) plot shows sample distribution according to DNAm levels in the top 25% most-variable CpGs (N=64,787). (**G-H**) Epigenetic age values measured by the Wagner epigenetic clock (G) and the Reik epigenetic clock (H) using mouse array data. (**I-J**) Values of proliferative history scores measured by the epiCMIT hypermethylation (I) and hypomethylation (J) epigenetic clocks. *p<0.05, **p<0.01, ***p<0.001.

**Figure 2.**
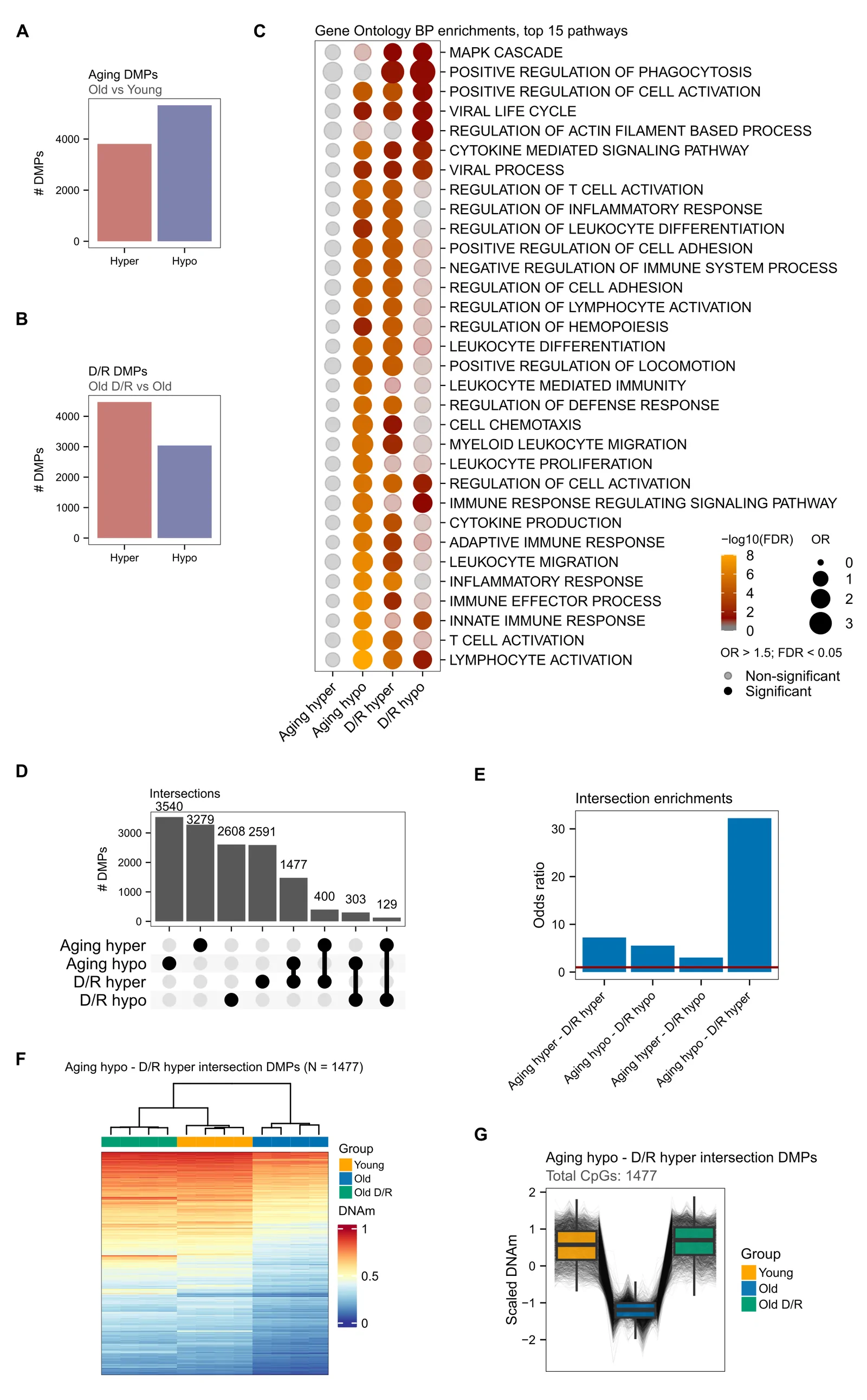
**Microglial repopulation reverses age-associated DNA methylation changes at the genome-wide level**. (**A-B**) Number of differentially methylated probes (DMPs, FDR <0.05, >5% methylation change) comparing old versus young subjects (A) and old-D/R versus old subjects (B). (**C**) Top-15 GO pathways significantly enriched (FDR<0.05, OR>1.5, >10 and <750 genes) for aging hyper- and hypomethylated DMPs and D/R hyper- and hypomethylated DMPs. (**D**) Upset plot indicating the intersections between aging hyper- and hypomethylated DMPs and D/R hyper- and hypomethylated DMPs. (**E**) Odds ratio (OR) enrichment of the intersection between the different sets of aging and D/R DMPs. The red line indicates OR=1. All intersections were enriched (Fisher’s test p<0.001). (**F**) Heatmap indicating the DNAm values of the 1,477 DMPs significantly hypomethylated with aging and hypermethylated with D/R. (**G**) Scaled levels of DMPs shown in (F).

Beyond the changes in specific genomic regions revealed by the epigenetic clocks, microglial D/R may induce other DNAm alterations, which could potentially explain its association with the reversal of aging phenotypes. Differential methylation analyses (FDR<0.05, >5% DNAm difference) comparing microglia from young and old mice, and microglia from old and old-D/R mice showed widespread DNAm alterations, with 9,128 differentially methylated probes (DMPs) being detected for aging; 58.3% displaying hypomethylation (Fig. 2A, see extended Zenodo dataset Supplementary Table 1). Conversely, 7,508 DMPs were detected in old-D/R microglia, the majority of which (59.5%) were hypermethylated (Fig. 2B, see extended Zenodo dataset Supplementary Table 1). Aging hypermethylation changes were not particularly enriched in CpG island-related locations (Fisher’s test p=0.5505, odds ratio [OR]=1.02, Supplementary Fig. 1A), although hypomethylation was enriched in open sea locations (Fisher’s test p<0.001, OR=1.43, Supplementary Fig. 1A). Microglial D/R-associated alterations were enriched at open sea locations for both hypermethylation and hypomethylation (Fisher’s test p<0.001 and <0.001, OR=1.57 and 2.95 for hyper- and hypomethylation changes, respectively, Supplementary Fig. 1B). Both aging and D/R-mediated alterations were particularly enriched at intronic regions for gains and losses of methylation (Fisher’s test all p<0.001, all OR>1.21, Supplementary Fig. 2 A-B). These observations imply that both aging and D/R induce considerable alterations to the microglial methylome that in global terms appear to go in opposite directions, and that these DNAm signatures manifest specific, nonrandom genomic distributions.

A gene set enrichment analysis of DMPs (FDR<0.05, OR>1.5) revealed widespread alteration of a common nexus of altered Gene Ontology (GO) pathways in aging and D/R (Fig. 2C, see extended Zenodo dataset Supplementary Table 2). Hypomethylation in aged microglia and repopulation-related hypermethylation were overrepresented in immunological pathways, including those related to immune activation, chemotaxis, and inflammatory response. Comparatively, D/R-induced hypomethylation showed subtler trends (Fig. 2C). These findings were also observed, particularly for aging hypomethylation, when screening the Hallmark database (Supplementary Fig. 3, see extended Zenodo dataset Supplementary Table 3). Therefore, inflammatory pathways suffer a general loss of DNAm in microglia during aging, while microglial D/R leads to gains of DNAm at these same gene sets. This finding is consistent with the exacerbated ischemia-induced inflammatory response showed by microglia in aged versus young mice, and the attenuated response after microglial D/R [7].

We then looked at the specific intersections between aging- and D/R-DMPs (Fig. 2D) finding that the largest group of shared alterations were between aging hypomethylation and D/R-induced hypermethylation. Although all the intersections were greater than expected by chance (all Fisher’s p<0.001), the aging hypomethylation-D/R hypermethylation intersection was by far the most overenriched (OR=32.2, Fig. 2E). This collection of 1,477 CpGs represented a set of genomic loci in which microglial D/R reverted aging-associated hypermethylation leading to old repopulated subjects displaying microglial epigenomic profiles more like young subjects (Fig. 2F-G). Gene set enrichment of these reversal DMPs again showed strong associations with immunological response pathways (Supplementary Fig. 4, see extended Zenodo dataset Supplementary Table 4), suggesting that the altered CpGs could be involved in microglial regulatory functions. Furthermore, using microglia enhancer location data from EnhancerAtlas [36], we found that the reversal DMPs were enriched at CpGs intersecting microglial enhancers (Fisher’s test p<0.001, OR=2.67, Supplementary Fig. 5) suggesting the targeting of regulatory elements [41].

## DISCUSSION

The epigenetic changes observed in aged microglia following D/R may underlie the functional benefits of this intervention in aged mice [7, 13, 14, 39]. Although some aging-related inflammatory signatures are not reversed by D/R under steady-state or inflammatory challenges [13, 14], inflammatory responses after acute brain damage are reduced [7, 15]. Our results reveal a complex pattern across immune pathways, including a partial reversal of age-associated hypomethylation in complement and interferon pathways, but not in others such as TNF-α signaling via NF-κB (Supplementary Fig. 3). A previous study showed that repopulated microglia in old mice upregulate genes linked to chromatin remodeling and epigenetic regulation (e.g., *Smarcad1*, *Actl6a*, *Gatad2a*, *Hdac2*, *Smarcb1*, *Myc*) [42], suggesting that epigenetic transitions may underlie their altered state. The identification of potentially reversible aging-associated traits in microglia may inform the development of strategies aimed at rejuvenating the aging brain and enhancing outcomes in age-related neurological diseases. However, future studies should further explore the functional consequences of increased proliferative history in repopulated microglia. Notably, our findings highlight the need for caution when interpreting epigenetic age as a direct measure of biological aging, since prior cell divisions can influence epigenetic clocks [43, 44]. Furthermore, our results should be interpreted in the context of the multifactorial mechanisms driving aging and neurodegeneration. As reported in the literature, factors such as genetic instability, epigenetic changes, iron accumulation, oxidative stress, impaired proteostasis and mitophagy, mitochondrial and lysosomal dysfunction, neuroinflammation and cell cycle deficits all contribute to age-related pathologies [45-48].

This study has certain limitations. Although both sexes were included, it was not designed or powered to detect sex effects. However, some responses to microglial D/R may be sex-dependent [49, 50]. Another limitation is that we did not assess functional outcomes of microglia D/R, although prior studies in aged mice report cognitive and sleep-wake improvements under steady-state conditions [13, 39], reduced neurological deficits after brain hemorrhage [15], and we found improved motor function following stroke [7]. Finally, we applied epigenetic clock models trained in other tissues and platforms, but the development of microglia-specific models will be important to validate the robustness of our findings.

Overall, this study shows that the epigenetic age of microglia can be assessed using epigenetic clocks. Moreover, stroke-induced brain injury or microglial D/R in old mice accelerates the epigenetic aging of microglial cells, which is attributable to increased microglial proliferation. Additionally, unlike original microglia from old mice that show hypomethylation across multiple GO pathways, repopulated microglia show hypermethylation in many of the same pathways, suggesting opposing functional outcomes. Therefore, our results suggest that microglial repopulation exerts contrasting age-related effects on the DNA methylome since it increases the epigenetic age yet reverses a substantial fraction of aging associated DNAm changes in microglia.

## Supplementary Materials

The Supplementary data can be found online at: www.aginganddisease.org/EN/10.14336/AD.2025.1066.

## Data Availability

The data underpinning this article are provided in the main text and Supplementary Material. Extended datasets, including the sets of differentially methylated probes and gene enrichment results, are accessible at Zenodo (10.5281/zenodo.17151265). Additionally, the raw methylation data (IDAT files) and preprocessed matrices are deposited in ArrayExpress under accession E-MTAB-15631.
