# Supplementary material for: Microglial Replacement Reverses Age-Associated Epigenetic Modifications Despite Accelerating Epigenetic Age
Source: Aging Dis. 2026 Oct 11;17(6):3442–52. doi: 10.14336/AD.2025.1066 (PMC13653709; doi:10.14336/AD.2025.1066)
Supplement: Supplementary file 1 [file AD-17-6-3442-s.pdf]

SUPPLEMENTARY DATA

Microglial replacement reverses age-associated epigenetic modifications despite accelerating epigenetic age

Maria Arbaizar-Rovirosa, Raúl F. Pérez, Alfonso Peñarroya, Mattia Gallizioli, Mario F. Fraga, Anna M. Planas

© 2025. Arbaizar-Rovirosa M et al. Published online at http://www.aginganddisease.org/EN/10.14336/AD.2025.1066

SUPPLEMENTARY DATA

This document contains the Supplementary Figures and Supplementary Table legends for the study by Arbaizar-Rovirosa M, Pérez RF et al.

Extended datasets (Tables S1-4), including the sets of differentially methylated probes and gene enrichment results, are accessible at Zenodo (10.5281/zenodo.17151265). Additionally, the raw methylation data (IDAT files) and preprocessed matrices are deposited in ArrayExpress under accession E-MTAB-15631.

SUPPLEMENTARY FIGURES

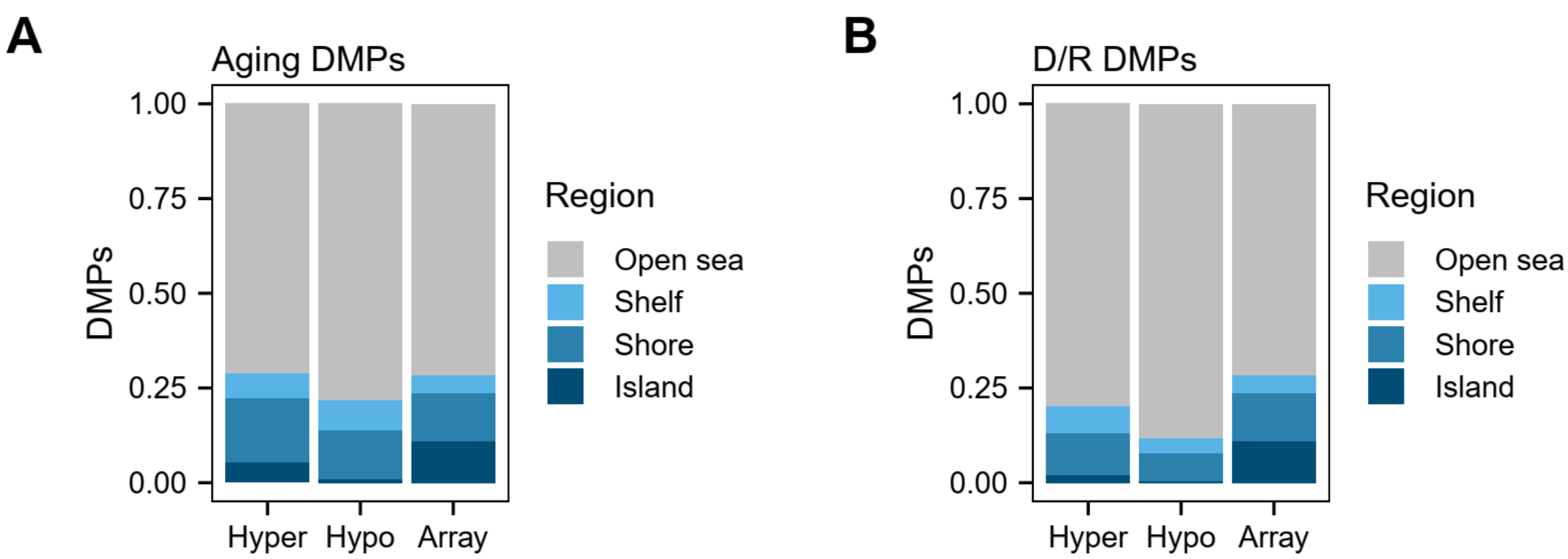

Supplementary Figure 1. A-B) Bar plots indicating the proportions of DMPs annotated to CpG island-related locations for hyper- and hypomethylated aging (A) and D/R (B) DMPs. The rightmost bar shows the background distribution of all measured array CpGs.

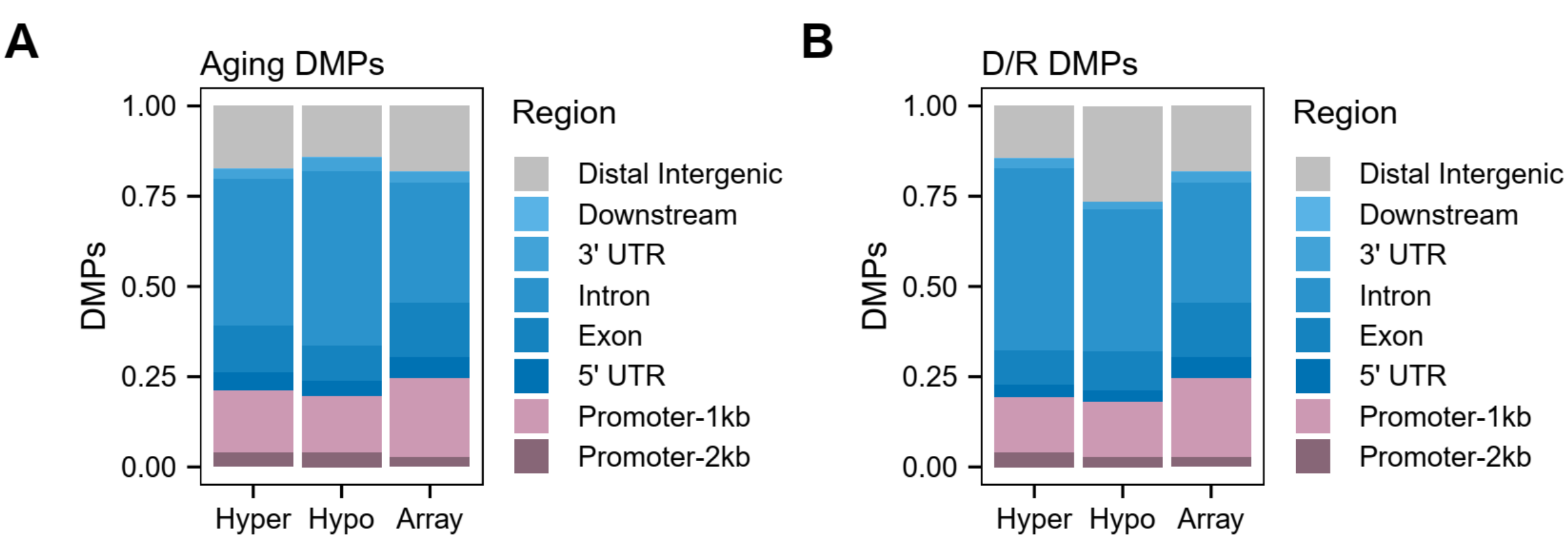

Supplementary Figure 2. A-B) Bar plots indicating the proportions of DMPs annotated to gene region-related locations for hyper- and hypomethylated aging (A) and D/R (B) DMPs. The rightmost bar shows the background distribution of all measured array CpGs.

© 2025. Arbaizar-Rovirosa M et al. Published online at http://www.aginganddisease.org/EN/10.14336/AD.2025.1066

SUPPLEMENTARY DATA

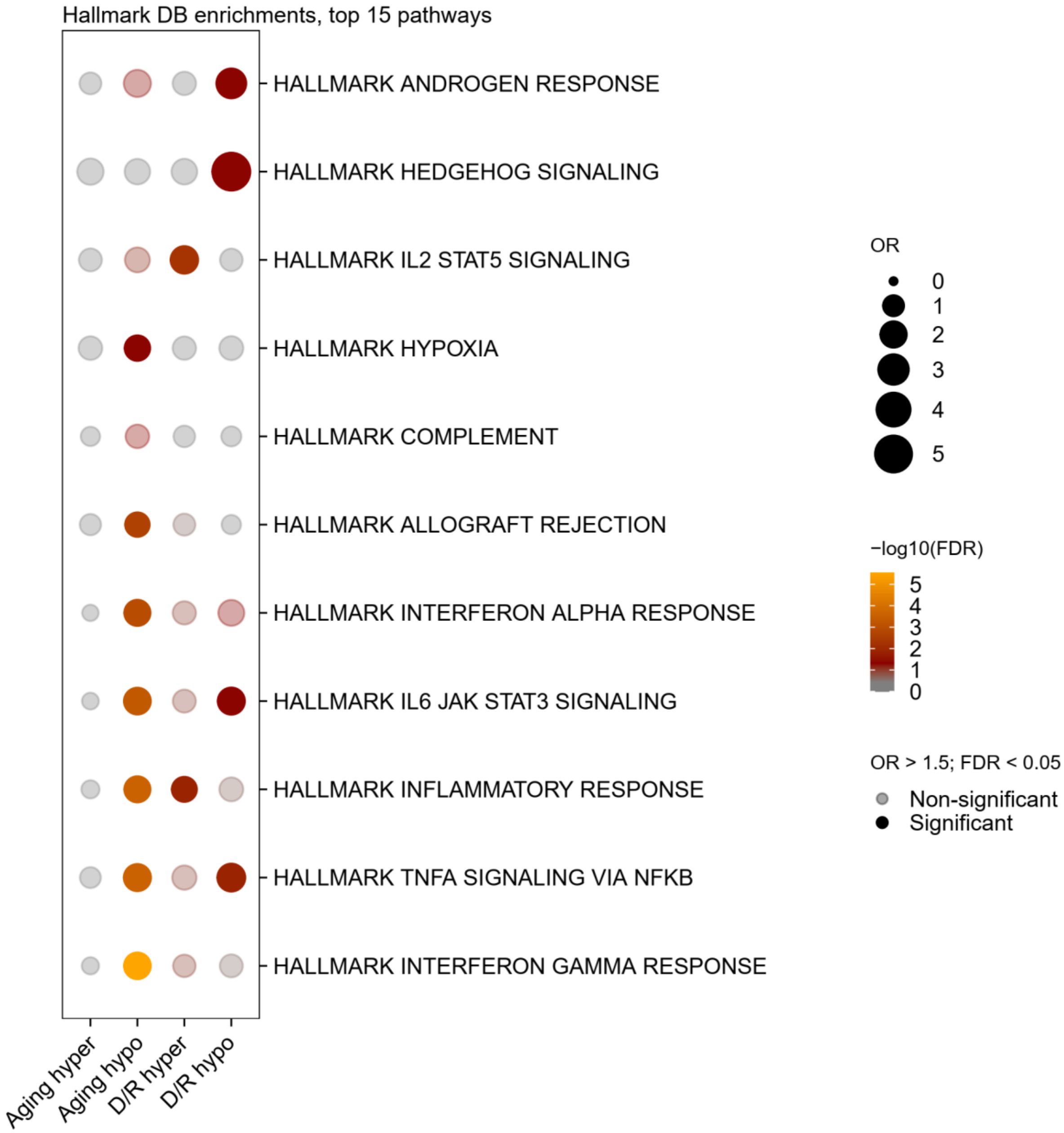

Supplementary Figure 3. Bubble plot showing the top 15 Hallmark Database pathways found significantly enriched (FDR < 0.05, OR > 1.5, at least 10 genes and less than 750 genes) for aging hyper- and hypomethylated DMPs and D/R hyper- and hypomethylated DMPs.

© 2025. Arbaizar-Rovirosa M et al. Published online at http://www.aginganddisease.org/EN/10.14336/AD.2025.1066

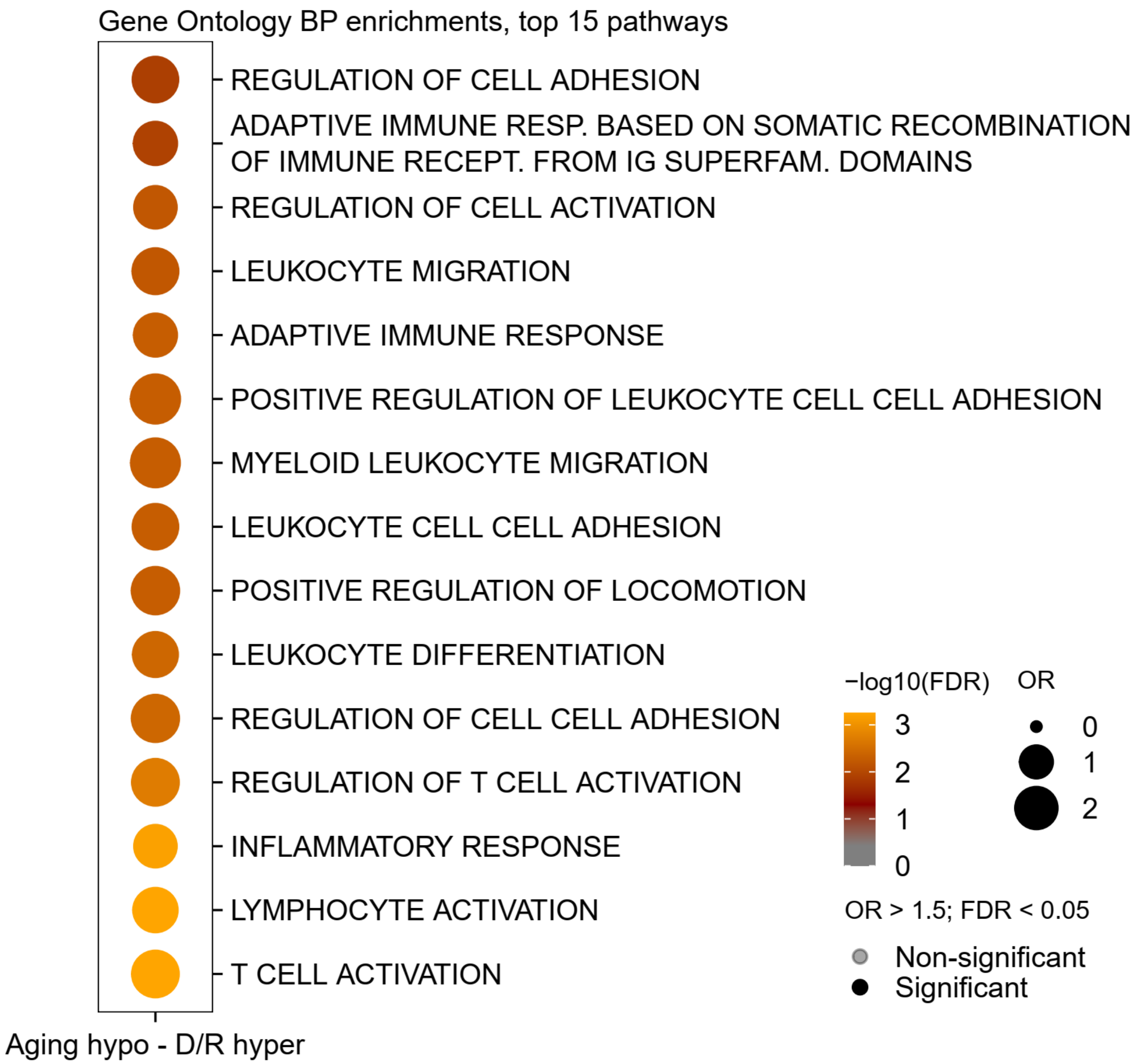

Supplementary Figure 4. Bubble plot showing the top 15 Gene Ontology Biological Process pathways found significantly enriched (FDR < 0.05, OR > 1.5, at least 10 genes and less than 750 genes) for the 1,477 DMPs which are significantly hypomethylated with aging and significantly hypermethylated with D/R.

© 2025. Arbaizar-Rovirosa M et al. Published online at http://www.aginganddisease.org/EN/10.14336/AD.2025.1066

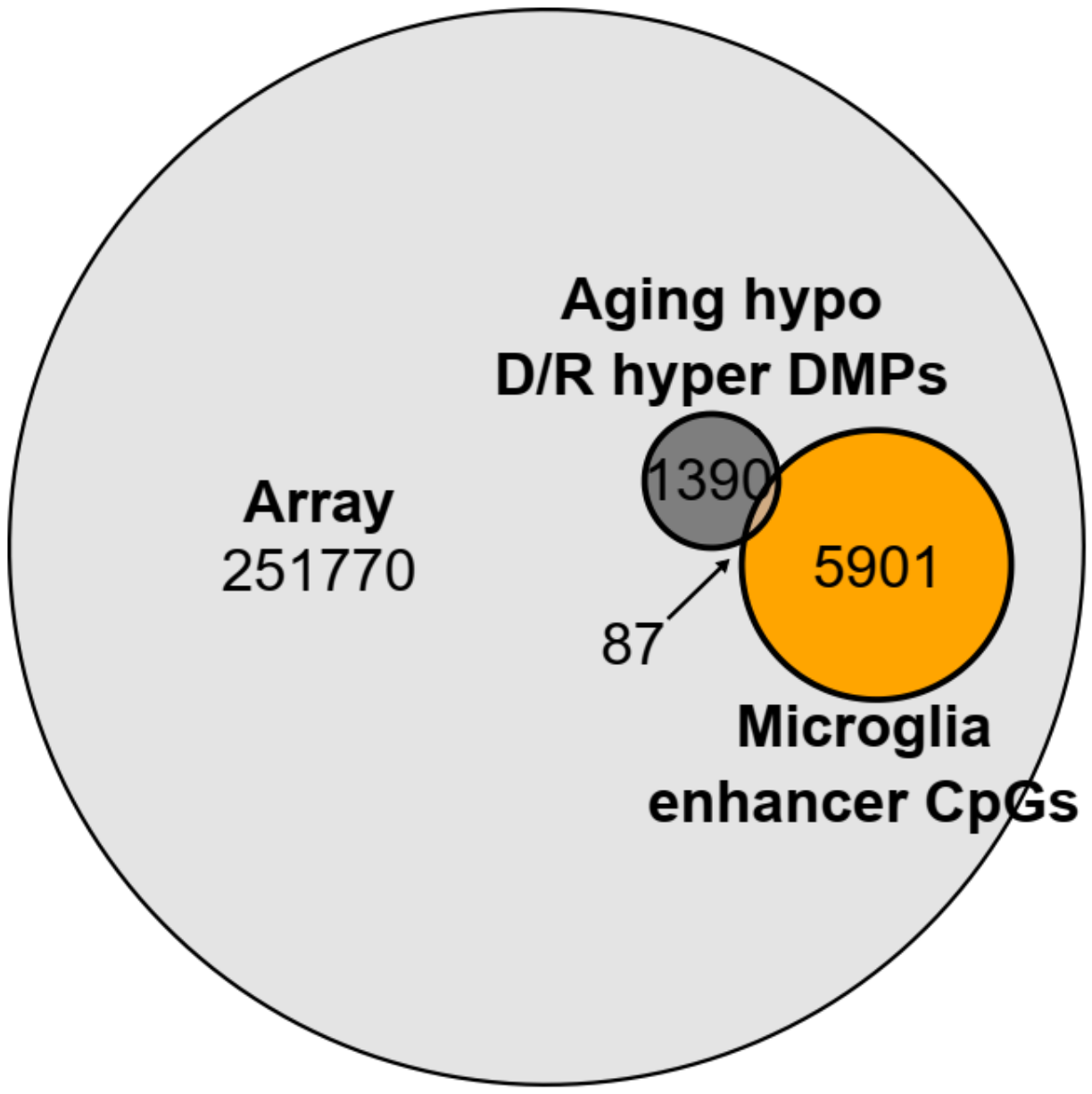

Supplementary Figure 5. Euler diagram representing the number of aging hypomethylation–D/R hypermethylation DMPs which intersect with microglia enhancer-associated locations. Set sizes are proportional, except for the array, which was scaled down for graphical clarity.

© 2025. Arbaizar-Rovirosa M et al. Published online at http://www.aginganddisease.org/EN/10.14336/AD.2025.1066

SUPPLEMENTARY DATA

Supplementary Tables at Zenodo (10.5281/zenodo.17151265).)

Supplementary Table 1. List of the differentially methylated probes (DMPs, FDR < 0.05, methylation change > 5%) found for the aging (old versus young) and D/R (old D/R versus old) comparisons.

Supplementary Table 2. List of the Gene Ontology Biological Process pathway enrichments results obtained when testing the genes associated with the differentially methylated probes (DMPs, FDR < 0.05, methylation change > 5%) found for the aging (old versus young) and D/R (old D/R versus old) comparisons.

Supplementary Table 3. List of the Hallmark Database pathway enrichments results obtained when testing the genes associated with the differentially methylated probes (DMPs, FDR < 0.05, methylation change > 5%) found for the aging (old versus young) and D/R (old D/R versus old) comparisons.

Supplementary Table 4. List of the Gene Ontology Biological Process pathway enrichments results obtained when testing the genes associated with the differentially methylated probes (DMPs, FDR < 0.05, methylation change > 5%) which intersect between aging hypomethylation and D/R hypermethylation.
